# Early Steps towards Hearing: Placodes and Sensory Development

**DOI:** 10.3390/ijms24086994

**Published:** 2023-04-10

**Authors:** Azel Zine, Bernd Fritzsch

**Affiliations:** 1 LBN, Laboratory of Bioengineering and Nanoscience, University of Montpellier, 34193 Montpellier, France; 2 Department of Biology, CLAS, University of Iowa, Iowa City, IA 52242, USA

**Keywords:** inner ear, gene regulatory network, epibranchial, sensory placode, hair cell, sensory neuron, taste buds

## Abstract

Sensorineural hearing loss is the most prevalent sensory deficit in humans. Most cases of hearing loss are due to the degeneration of key structures of the sensory pathway in the cochlea, such as the sensory hair cells, the primary auditory neurons, and their synaptic connection to the hair cells. Different cell-based strategies to replace damaged inner ear neurosensory tissue aiming at the restoration of regeneration or functional recovery are currently the subject of intensive research. Most of these cell-based treatment approaches require experimental in vitro models that rely on a fine understanding of the earliest morphogenetic steps that underlie the in vivo development of the inner ear since its initial induction from a common otic–epibranchial territory. This knowledge will be applied to various proposed experimental cell replacement strategies to either address the feasibility or identify novel therapeutic options for sensorineural hearing loss. In this review, we describe how ear and epibranchial placode development can be recapitulated by focusing on the cellular transformations that occur as the inner ear is converted from a thickening of the surface ectoderm next to the hindbrain known as the otic placode to an otocyst embedded in the head mesenchyme. Finally, we will highlight otic and epibranchial placode development and morphogenetic events towards progenitors of the inner ear and their neurosensory cell derivatives.

## 1. Introduction

Ear and epibranchial placodes are originating from the ectoderm to generate neurons and sensory cells, including the underlying gene regulatory networks, and signaling pathways to make sensory neurons, hair cells and taste buds. Much early development has been characterized in animal models, mostly in mice, but it is incompletey described for humans, including CHARGE [1,2] and BOR [3,4,5] syndrome defects.

Hearing starts from the placode that will be transformed into the otocyst or otic placode. The prosensory domain of the otic placode gives rise to the vestibular system as well as the cochlea and the spiral ganglion neurons (SGNs) that will connect with the cochlear hair cells [6,7]. Once the connection between the SGNs and the cochlear nuclei is established, the central auditory system develops to reach out the inferior colliculi and the auditory cortex [8,9]. In contrast to the gain of hair cells, SGN, cochlear nuclei and the auditory system, we also have a decline of sensory and neuron losses that will reduce the sound input [10,11]. Hearing deficits will likely affect close to one billion people [12] who are living with Alzheimer’s disease [13,14,15]. Likewise, the associated epibranchial placode that gives rise to distinct neuron development [16,17] can lose the sensory cells of taste in aging people [18,19,20]. Human placode development and restoration has remained a serious medical condition caused by the dysfunction of placode-derived tissues. The formation of new hair cells, vestibular ganglion neurons (VGN), SGNs, epibranchial neurons, taste buds, brainstem and cortex are a common problem in humans for which we need a solution to generate new sensory receptors and neurons.

In the context of the inner ear, cell-based strategies to replace damaged neurosensory cells by implantation of different cell types into the inner ear represent a challenge which is in continuous progress [21,22]. Transplantation of stem cell-derived otic neuronal and epithelial progenitor cells into the cochlear nerves or into the cochleae has been demonstrated in animal models of SNHL. Successful engraftment and integration have been observed into the target sites, although with a lower survival rate, and displayed molecular features of early neurosensory differentiation [23,24,25,26].

The results from these studies are a proof-of-principle, that transplantation of partially differentiated otic progenitors may be a useful for cell-based cell therapy therapeutic strategy to treat SNHL.

Despite significant progress in inner ear transplantation, methodologies will need to be refined to generate a homogenous population of differentiated otic progenitor cells in 2D and 3D culture systems and their optimal molecular characterization before in vivo implantation.

Stem cell-derived inner ear organoids are also in continuous progress for the generation of neurosensory-like cells. They harbor fairly ordered tissues closer to those in the in vivo developing ear than can be achieved in the 2D monolayer culture system, allowing for increased recapitulation of some developmental events. In these stem cell-derived 3D cultures, otic vesicles form autonomously within the aggregates, following otic–epibranchial progenitor domain formation [27,28]. In a similar manner, initial cell culture studies with isolated otic placodes have shown that once induction and invagination is complete, sensory cell and neuronal differentiation is autonomous [29]. However, in the current otic differentiation models, major limitations of the 3D organoid cultures are related to the variable reproducibility of the system [30,31], and the requirements to optimize protocols specific for a given pluripotent stem cell line [27,32] have substantially limited the studies using 3D inner ear organoids.

Therefore, a deep comprehension of the earliest requirements for pre-placodal ectoderm, epibranchial placode and sensory organ development is of utmost clarification to refine human 3D otic cell differentiation protocols that will contribute to the next generation of cell-based therapeutic approaches to restore sensorineural hearing loss.

In this review, we describe how the neural plate forms to generate the ear and epibranchial placode. We will also highlight the otic and epibranchial placode development towards progenitor cells and their neurosensory cell derivatives including hair cells and taste buds. 

## 2. Early Ear Placodal Development: From Unspecified Cells to Neural Plate and Pre-Placodal Ectoderm

**Figure 1:**
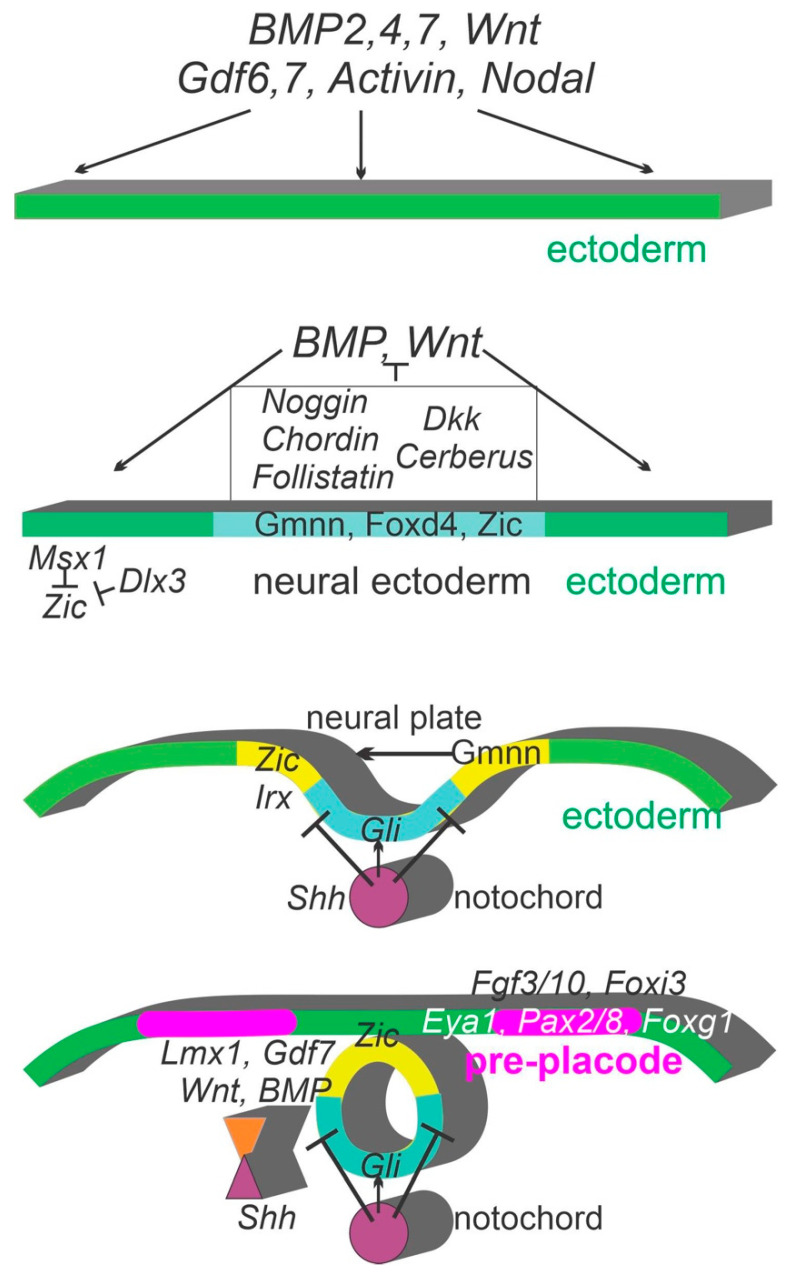
Comparison of the critical steps identified in distinct steps of neural induction of pre-placode. Initially, *Nodal*, *Activin*, *BMP* and *Wnt* are expressed throughout the ectoderm. *Gmnn*, *Zic* and *Foxd4* become expressed, upstream from *Irx,* and upregulate *Sox* expression. An early expression of *Shh* drives *Gli* expression ventral and is counteracted by the roof plate/choroid plexus. *Lmx1a/b* drives the dorsal expression of *Wnts* and *BMPs*, including *Gdf7*. Additional downstream genes of *Fgf3/10* and *Foxi3* are needed for the earliest pre-placode definition. Downstream and largely independent include genes such as *Eya1 and Pax2/8*, among others. Modified after [37,38,44,46,47,48,49].

With the earliest transformation from a common ectoderm into two major placodes, the otic and the epibranchial placodes share for the initial formation, but it also has a slightly different gene signaling cascade. Neural induction involves genes known to be important for the earliest steps to general body patterning that will generate the neural plate [33]. Initially, *BMP*, *Wnt*, *Activin* and *Nodal* are expressed throughout the ectoderm [34,35]. The transition from ectodermal tissue to neural ectoderm requires the downregulation of *BMP* by *Noggin*, *Chordin* and *Folistatin* (Figure 1) and the downregulation of *Wnt* by *Dkk* and *Cerberus* [36]. *Fgfs* also contribute to this process [34].

Following initial neural induction, at least three genes, *Geminin (Gmnn)*, *Zic* and *Foxd4* (in frogs), are expressed that further define and stabilize the neural ectoderm by inhibiting BMP and Wnt signaling as well as upregulating other pro-neural genes [34,35,37,38] (Figure 1). Overexpression of *Gmnn* leads to expression of *Zic1* and, while there is no direct interaction of these two genes, they were shown to have overlapping and cooperative roles in neuronal progenitor formation and maintenance [37]. Similarly, *Foxd4* promotes the proliferation of neuronal precursors [35] and regulates expression of the *Gmnn* and *Zic* genes [34,39]. 

The neural plate is induced following the inhibition of the epidermally expressed *BMPs* and *Wnts* along with the upregulation of the *Gmnn*, *Foxd4* and *Zic* genes [34,37,40]. The expression of *Sox* genes results in the transformation of these precursor cells into neural plate stem cells [34]. The *SoxB1* family (*Sox1*, *Sox2*, *Sox3*), together with *Gmnn*, *Foxd4* and *Zic2*, are essential for the continued proliferation of undifferentiated neural stem cells [34]. The explosive proliferation of these neural stem cells and the coordination with convergent–extension leads to the folding of the neural plate into the neural tube (Figure 1; Refs. [40,41]). The following transition from a neural stem cell into a neuronal progenitor cell requires the expression of *Sox11* [34]. For a neuronal progenitor cell to exit the cell cycle and proceed to differentiate, the initial upregulation of *Gmnn*, *Foxd4*, *Zic*s and *Sox* genes must be downregulated [37,38,42,43]. Following the downregulation of the various proliferative genes, bHLH genes involved in neuronal differentiation in the CNS become expressed in dorso-ventral expression patterns within the developing neural tube to regulate fates of neurons within various subdomains [44,45]. 

After the neural plate fuses to form the neural tube (Figure 1), the newly formed dorsal roof plate begins to express *BMPs* and *Wnts* while the ventral floorplate and underlying notochord expresses *Shh* [50,51]. Although many genes play a role in patterning the neural tube, *BMPs*, *Wnts* and *Shh* play a primary role [50]. Expression of *Wnts* and *BMPs* in the dorsal neural tube is driven by the dorsal expression of *Lmx1a/b* [44,46,52]. *Lmx1a/b* expression itself is in the roof plate and is regulated by *Zic1*, *Zic3* and *Zic4* [53]. While the timing of *Wnt* and *BMP* expression during neural tube formation varies between mice and frogs [38,54], these two genes, as well as *Fgfs* and retinoids, are critical in defining the dorsal part of the brainstem and spinal cord [55]. Loss of Wnt or BMP signaling negatively affects dorsal progenitor cells [46]. 

Determination of dorso-ventral identity within the brainstem is governed by the expression of various homeodomain and bHLH transcription factors in restricted areas along the dorso-ventral axis [45,55]. The dorsal brainstem is subdivided into eight domains of neuronal progenitor populations [55]. The bHLH gene, *Atoh1*, is expressed in the dorsal-most domain (dA1) throughout the brainstem and spinal cord [55,56]. *Atoh1*-positive cells generate the rhombic lip of the hindbrain, superficial migratory neuron streams and cerebellar granule cells, that contribute to auditory, vestibular, solitary tract and proprioceptive networks [57]. As expected from the expression pattern, loss of *Atoh1* eliminates most cochlear nuclei neurons [58]. Additional genes are expressed in the *Olig3* domain including *Neurog1/2* (dA2), *Ascl1* (dA3-dB1) and *Ptf1a* (dA4-dB1) [45,59]. For example, the dorsal-most subdomain (dA1), that also expresses *Atoh1*, expresses *Pax3*, the next subdomain (dA2) expresses *Pax3* and *Pax7* and the third subdomain (dA3) expresses *Pax3*, *Pax6* and *Pax7* [55]. Additional genes are uniquely expressed in progenitor subdomains, such as *Barhl1* in dA1 progenitors, *Lhx* in dA2, *Tbx* in dA3, *Foxd3*/*Foxp2* in dA2 and dA4 and *Phox2b* in dB2 [55]. Genes, such as *Pou4f1*, are expressed in multiple subdomains (dA1-dA3, dB3) [55]. Interestingly, *Atoh1*, which defines the dorsal-most progenitor population (dA1), is also expressed in a more ventral subpopulation (dB2) during their maturation [45,55,60]. *Phox2b* is expressed in rhombomere 2–6 of the hindbrain (dB2), but not in rhombomere 7 or the spinal cord [55]. This unique hindbrain domain (dB2) also later expresses *Atoh1*. A second unique domain in the hindbrain (dA4) expresses *Ptf1a*, *Foxd3* and *FoxP2,* among other genes [55]. Loss of *Ptf1a* results in the loss of dA4 and dB1 neurons in combination with the expansion of dA3 and dB3 neurons, leading to the eventual misspecification of somatosensory and viscerosensory nuclei neurons in the hindbrain [61,62]. A combination of bHLH genes *Ascl1*, *Neurog2* and *Olig3* defines dA3, in the brainstem, which extends through the spinal cord (rostral to caudal) at a location which includes the developing solitary tract [55]. In situ hybridization showed co-expression of *Rnx* (*Tlx3*) and *Phox2b* in the neurons of the solitary tract; in the absence of *Neurog1*, non-taste ganglia are lost. Projection neurons of nST are entirely absent in both *Tlx3 (Rnx) and Phox2b* knockouts, indicating that these neurons are dependent on these factors for their development [63].

Transition from neuronal progenitor to neuronal differentiation requires the interaction of pro-neural bHLH genes and additional bHLH genes, the Class I *Hes/Hey* genes [42] and the Class V *ID* genes [64,65,66]. Proliferation of neurosensory precursors is driven by *Hes*, *ID*, *Sox2* and *Myc* genes, and the transition to differentiated cells is controlled by the balance of Notch signaling molecules and the pro-neural bHLH genes. Oscillation of a pro-neural bHLH gene and a repressive one, such as *Ascl1* and *Hes1*, through cross regulation of each other controls the timing of neurogenesis [42]. This oscillation maintains proliferation of neuronal progenitors, whereas the eventual loss of *Hes1* expression and subsequent sustained expression of a pro-neural bHLH gene leads to neuronal differentiation [42]. The expression of downstream bHLH genes, such as *Neurod1*, adds to these complex interactions. For instance, *Atoh1* expression extends along the roof plate to the cerebellum, parallel to the slightly more ventral expression of *Neurog1/2*, and the loss of dorsal neurons in *Atoh1* null mice [57,58] results in a reduced cerebellum and auditory nuclei [52]. *Neurod1* negatively regulates *Atoh1* expression during cerebellum, and gut proliferation and manipulating *Neurod1* expression that may help to counteract Medulloblastoma [67,68,69,70]. *Atoh1* shows a much higher level of expression in the auditory nuclei and counteracts with *Neurod1*, indicating a differential regulation of expression in auditory nuclei [71]. 

In summary, an overview is provided of the neural placode that develops to generate the pre-placodal induction (Figure 1) and develops the hindbrain region that defines the different longitudinal expression of bHLH and other gene expression.

## 3. Patterning of the Pre-Placode Epithelium and Restriction of Pan-Placodal Development

Once the neural plate is fused and generates the roof plate, neural crest and placodes develop from immediately adjacent ectodermal tissue [33,72]. Neural crest and placodal neurons give rise to the formation of all or part of the various sensory systems. The work of Northcutt and Gans provided a novel perspective of the organization of the neural crest and placodes [73]. Development of neural crest and placodes is highly conserved across vertebrate species [33,74].

**Figure 2:**
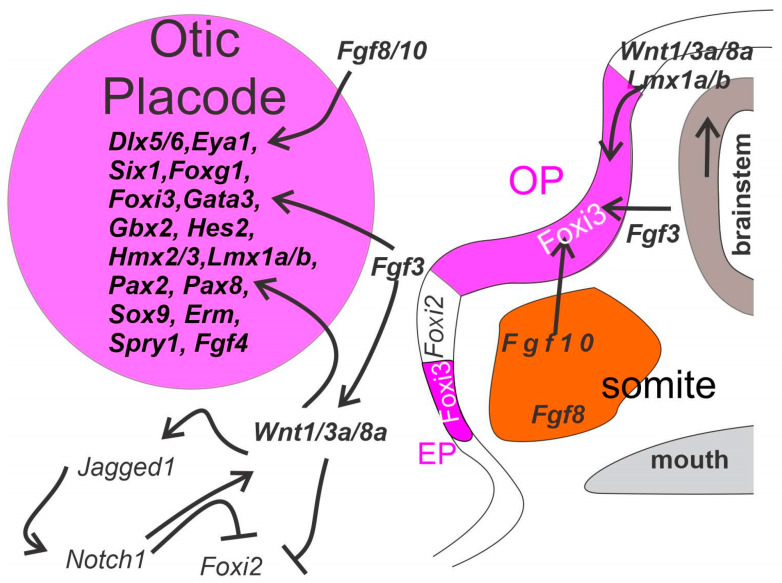
Otic placode (OP) and epibranchial placode (EP) induction. Fgf3 and Wnt1/3a/8a from the hindbrain downstream of *Lmx1a/b* as well as *Fgf8* and *Fgf10* from the surrounding somite’s combined with *Foxi3* are believed to be sufficient to induce the otic and epibranchial placode. The subsequent mechanism is not fully elucidated but may involve *Jagged1* and *Notch1* inhibition of *Foxi2*. Upregulation of *Dlx5/6*, *Eya1*, *Foxg1*, *Gata3*, *Gbx2*, *Hes2*, *Hmx2/3*, *Lmx1a/b*, *Pax2/8*, *Six1*, *Sox9*, *Spry1* and others marks the otic and epibranchial placode and is essential for later otic vesicle specification and morphogenesis. *Foxi2* delineates the ectoderm. Adapted with permission from Refs. [48,49,72,79].

Cranial placodes (Figure 1 and Figure 2) develop from a pan-placodal region at the neural border zone and give rise to the anterior pituitary gland, the olfactory epithelium, the lens of the eye and the large neurons in the trigeminal, facial, glossopharyngeal (petrosal) and vagus (nodose) nerves. This is in addition to the inner ear that contains the vestibular and auditory epithelia and sensory neurons [33,74]. The pan-placodal region broadly expresses *Eya1*, *Six1* and *Six4* [4,75]. During development, the pan-placodal region begins to subdivide in the trigeminal placode, the ear placode and three epibranchial placodes [16,76]. Differential expression of transcription factors along the anteroposterior axis gives rise to anterior and posterior compartments, which are further subdivided into individual placodes [77]. The posterior compartment gives rise to the otic and epibranchial placodes which expresses *Pax2*, *Pax8*, *Sox2* and *Sox3* [78].

While the vestibular system is highly conserved across vertebrates, the auditory system is much more variable [80,81]. Both sensory systems are housed within the inner ear. The otic placode uniquely depends on *Foxi3* [48] and *Fgf3/10* [49], but shares the requirement of *Eya1/2/Six1/2* with many other sensory placodes [82,83]. Additionally, *Foxg1* [84], *Lmx1a/b* [52], *Pax2/8* [85], *Fgf8* and *Fgfr2b* [86,87,88,89,90], *Gbx2* [91], *Gata3* [92,93] and *Shh* [94] are required for inner ear development (Figure 2). For instance, the ear never develops beyond the otocyst stage with the loss of both *Pax2* and *Pax8* [85] or *Eya1* deletion [4]. During development, the inner ear is patterned anteroposteriorly, dorsoventrally and proximal-distally [95]. *Shh* specifies the ventral portion of the ear, and in its absence, ventral structures, such as the cochlea, are absent [94,96,97]. In contrast, *BMP4* specifies dorsal vestibular structures [98,99]. Other genes, such as *Foxg1* [84,100], *Lmx1a/b* [101,102], *Otx1/2* [91,103] and *n-Myc* [104,105], affect specific sensory epithelia and/or structures of the inner ear. Notch signaling and retinoids are also involved in inner ear patterning [42,106,107].

Inner ear neurogenesis requires the induction of *Eya1* by *Sox2*, which in turn downregulates *Sox2* expression by *Neurog1* [108] that provides a negative feedback. *Neurog1* [109,110] upregulates *Neurod1* [8,111,112] and several other bHLH genes [113,114]. *Pou4f1* is also involved in neuronal development and in addition, for the proper pathfinding of inner ear afferent neurons [115,116]. Additional genes, such as *Npr1*, *Prickle1*, *Fzd3/6*, Ephrin’s and *Vangl2,* have been implicated in inner ear afferent central and/or peripheral pathfinding [117,118,119,120,121,122]. Neural crest induction occurs at the lateral edge of the neural plate [123]. Following closure of the neural tube, neural crest cells migrate throughout the embryo, contributing to a wide range of tissues including neurons, craniofacial skeleton, smooth muscles and melanocytes [72,124]. *BMP*, *Wnt*, *Fgf* and *Notch* signaling are all essential for the formation of neural crest cells [124]. Upon induction of the neural crest, many crest-specific genes are expressed, including *Sox5*, *Sox8*, *Sox9* and *Sox10* [72,123]. While there are specific species differences in the onset and sequence of expression, these *Sox* genes are important for the specification, migration and differentiation of neural crest cells [123,125]. Additional genes that are upregulated include *Snail*, *Slug*, *Pax3/7*, *Hairy2*, *Msx1/2*, *Dlx5* and *Gbx2* [72,124].

In summary, the earliest induction of distinct derivatives for the formation is presented of pre-placodal to pan-placodal development.

## 4. Development of Distinct Placodal Derivatives of the Otic Placode and the Epibranchial Placode

**Figure 3:**
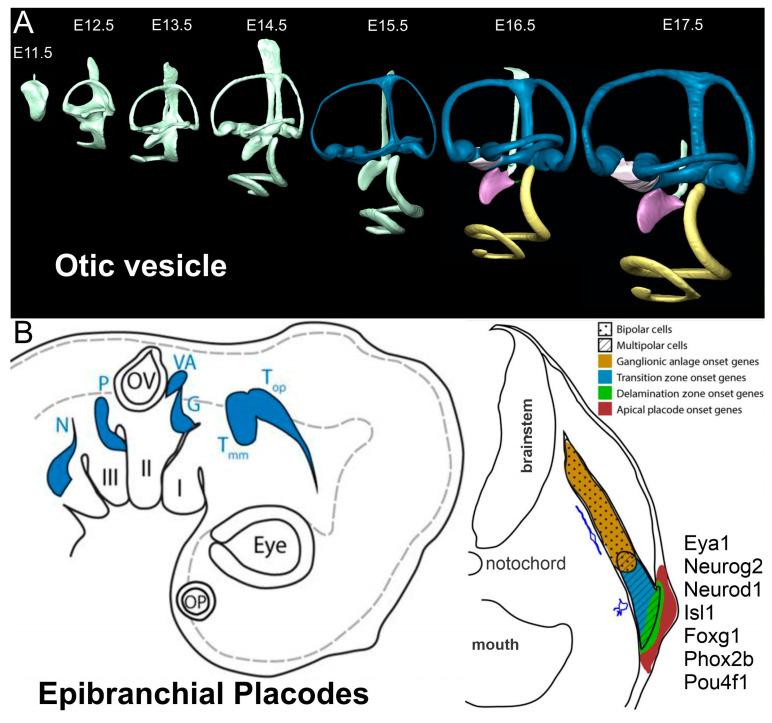
Generating the otic vesicle and the epibranchial placode. (**A**) The otic vesicle is depicted in a mouse using 3D reconstruction of the ear. An early formation segregates from the endolymphatic duct (white). Separate color is defined as the canal cristae (blue) next to the utricle (white) that interconnects the saccule (lilac). Note the progression of the length of the cochlear duct can about E16.5 (yellow). (**B**) The neuronal differentiation of epibranchial placodes starts with *Eya/Six* next to the otic vesicle (OV) followed by *Pax2* and *Sox2*, which initiate transformation of the geniculate (G), petrosal (P) and nodose (N). As neuroblasts migrate from the ectoderm to deeper locations, additional factors are expressed in sequence, *Neurog2* (in mice, *Neurog1* in chicken) followed by *Neurod1*, *Isl1*, *Foxg1*, *Pou4f1* and *Phox2b*. Abbreviations: OP, olfactory placode; OV, otic vesicle, T, trigeminal neurons; VA, vestibular and auditory neurons. Reprinted with permission from Refs [17,105,128].

A unique feature is forming by sinking in the placode to generate an otic cup (Figure 2; about E9 in mice, d24 in humans) that will pinch off to form an otic cyst (about E9.5 in mice, d26 in humans). At E10 in mice (d28 in humans), the otocyst is separate from the overlaying ectoderm. The next step is to develop an endolymphatic duct that elongates. Caveating of the otocyst will develop the three semicircular canals, the utricle and saccule and elongates to become the cochlear duct (Figure 3, Ref. [105]). In contrast, only neurons are generated from epibranchial placodes [16,126]. Specifically, the facial (VII), glossopharyngeal (IX) and vagal (X) neurons develop from placodal and neural crest cells [127]. These neurons project centrally to specific brainstem nuclei (solitary tract, VII, IX, X; Figure 3) and peripherally to sensory cells (taste buds).

Induction of the epibranchial placodes depend critically on the expression of *Eya1/2/Six1/4*. These genes are upstream of *Pax2*, which help define the caudal (*Pax2/8*, VII, IX, X) placodes [75,126]. Additional genes are important in the development of the placodes contributing to the different cranial ganglia. For instance, *Neurog1* is necessary for trigeminal sensory ganglia [129], whereas *Neurog2* is necessary for sensory ganglia that develop from epibranchial placodes (VII, IX, X) [130]. Downstream of *Neurog1/2* are *Neurod1*, *Isl1* and *Pou4f1* genes, which promote the differentiation of placodally derived sensory neurons [111,115,129,130,131]. In addition, the epibranchial placodally derived neurons (VII, IX, X) depend upon *Phox2b* expression [17], which is downstream of *Eya1/Six1* [82]. Additional genes such as *Notch*, *Hes*, *Rbpj*, *Fgf8*, *PDGF* and *Wnts* are important for proliferation and differentiation of neurons [132].

In summary, we provide an overview of the placodal development that will incite future interactions to make different sensory neurons of the ear and epibranchial neurons first followed by the hair cells and taste buds that are forming later.

## 5. Hair Cells of the Inner Ear Have a Shared Developmental Program of Neurons

**Figure 4:**
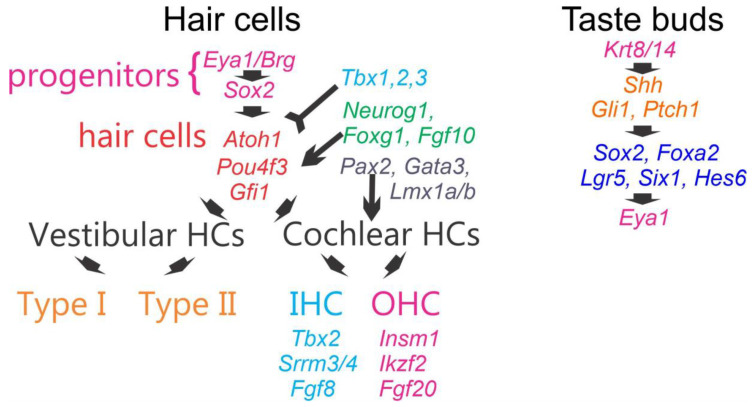
Hair cells and taste buds are independently derived. Progenitors of hair cells depend on *Eya1*, *Sox2* and *Atoh1* that are needed for their development. Downstream are *Pou4f3* and *Gfi1* that are needed to maintain hair cells. *Tbx1*,*2*,*3* interacts with *Neurog1,* and *Foxg1* regulates the number and distribution of cochlear to make it shorter and has increased the number of hair cells. A loss of all cochlear hair cells is revealed in *Pax2*, *Gata3* and *Lmx1a/b* mice. Two types of vestibular hair cells exist that have a mixed distribution of type I and type II HCs. In contrast in the cochlea, we have a single row of IHCs and three rows of OHCs. *Tbx2*, *Srrm3/4* and *Fgf’s* are needed for differentiation and viability of IHC. *Insm1*, *Ikzf2* and *Fgf20* are needed to differentiate OHCs or requires for forming three rows of OHCs. A very different sequence of genes is needed in taste buds. Starting is the upregulation *Krt8/14* that is prior to *Shh*. Downstream is *Sox2* that is required for taste bud differentiation. Overlapping are other genes some of which seem to differentiate into distinct taste sensory input. Compile with permission from Refs. [4,8,16,52,84,110,140,141,142,143,144,145].

Vestibular hair cells generate at least two types, type I and type II (Figure 4 [76,133,134]). A unique development is defined for the mammalian cochlear hair cells, the inner hair cells (IHCs) and outer hair cells (OHCs). Upstream is the expression of *Eya1* and *Brg1* that are needed for the initiation of hair cell progenitors. Downstream is the expression of *Sox2* to interact with specific bHLH genes to initiate cochlear hair cells [108]. The bHLH gene *Atoh1* (Figure 4) is needed for vestibular and cochlear hair cell formation beyond undifferentiated progenitor hair cells [6,135]. Downstream are *Pou4f3*, *Gfi1* and *Barhl1* gene expression that are needed for hair cell maintenance [136].

An interaction is existing between the bHLH gene *Neurog1 and Foxg1* that causes a reduced expression of *Atoh1*: The absence of *Neurog1* or *Foxg1* results into a short and wider cochlear set of hair cells that leads to more rows of OHCs (instead of three rows of OHCs; [84]). A misexpression of *Neurog1* instead of *Atoh1* results in IHCs and OHCs that are not functional and are converted as inner pillar cells into gaps between near normal IHCs [137]. An interaction also shows a shorter cochlea and converts OHCs into IHCs after deletion of *Neurod1*, a bHLH gene. Proliferation of more hair cells depend on n-*Myc* for normal differentiation [105]. Most recently, an effect of follistatin (*Fst*) is found in the apex [138]. Combined, the cochlear apex and base are differentially affected by *Lmx1a*, n-*Myc*, *Fst*, *Neurog1* and *Foxg1*, among others.

Downstream of OHCs depend on *Insm1* and *Ikzf2* that are needed for OHC development; OHC development interacts with *Neurod1* that also regulates OHCs instead converted to IHC [139]. In contrast, loss of *Tbx2* converts IHC into OHC-like hair cells [140]. Furthermore, IHC depends on *Fgf8* and *Srrm3/4*, and cochlea will lose nearly all IHCs in the absence of these two selectively expressed genes (Figure 4; Ref. [141]). 

Several genes are needed for the cochlear development which results in the absence of all cochlear hair cells. In the absence of *Gata3*, *Lmx1a/b and Pax2* (Figure 4), the cochlea do not develop any hair cell that likely interacts with *Atoh1* for hair cell formation [6,52,85,93]. In addition, we have a delayed loss of all hair cells that initially develop after a loss of *Bdnf/Ntf3* double deletions [146]. Moreover, *Bdnf* deletion will result in the loss of apical OHCs, while the loss of *Ntf3* causes the loss of OHCs in the basal cochlear turn. Likewise, it is dependent and will degenerate in *Cdc42* for IHCs first, followed by OHCs in a base to apex progression [147,148,149]. In addition, *MANF* deletion results in the loss of OHCs in the base of the cochlea [150]. 

There is a delay between SGNs and cochlear HCs: SGNs form first in the base (E10.5) to apex (E12.5). In contrast, cochlear HCs start to from in the apex around E12.5 and progresses to the base on E14.5 [110]. *Atoh1* is needed for all HCs but has a different progression starting in the base at E14.5 and progresses to the apex around E18.5 [135]. Innervation is reaching IHCs starting at E15 that expand to reach OHC innervation by Type II fibers at E18 (Figure 4; Ref. [151]).

In summary, at least two types of cochlear HCs develop that have a unique input–output connection [7,152] that are likely unrelated to the types I and II of vestibular hair cells [6,134].

## 6. A Unique Set of Genes Is Needed for Taste Bud Development

Like the taste ganglia, taste bud development is orchestrated by a specific sequence of gene expression and trophic interactions with nerve fibers. In the tongue, taste buds are in the papillae, which develop from placodes that arise prior to innervation and taste bud formation [153,154]. The initial signals that orchestrate taste bud development and establish their patterns on the tongue arise from the tongue epithelium [154,155]. Interestingly, signal patterning of the location of taste buds differs depending on the tongue region (i.e., fungiform papillae vs. circumvallate papillae), likely because these tissues arise from different branchial arches. Before any other gene is expressed, the earliest expression is *Krt8* followed by *Krt14* [143]. Specifically, fungiform papillae (Figure 4) are patterned during development by sonic hedgehog (*Shh*) and Wnt signaling pathways [156,157,158,159], whereas the circumvallate papilla is regulated by fibroblast growth factor 10 (*FGF10*) and its receptors *Spry1-2* [88,160]. The cells within the developing taste epithelial placode that express *Shh* differentiate into taste buds during development [161]. Prior to differentiation, these *Shh*+ placodal cells become innervated, and this innervation is required to maintain *Sox2* expression [162]. Both *Sox2* expression and innervation are required for continued taste bud development [145,163]. A differentiation requires *Lgr4*, *Six1*, *Hes6* and *Foxa2* expression [143,164], among others. The loss of taste bud innervation following the knockout of either the neurotrophin, *BDNF*, or its receptor, *TrkB*, results in a loss of taste buds over time [165,166,167]. The factors that are produced by the neurons to support continued taste bud development are unclear; however, *Shh* and R-*spondin* are likely possibilities [168,169,170]. Although the taste system matures, some developmental processes continue into adulthood, including taste bud cell differentiation. One unique feature of taste buds is that cells are replaced every 8–20 days, depending on the specific taste bud types [171,172,173]. The stem cells that give rise to adult taste buds express *Lgr6* in the fungiform papillae, but express *Lgr5* within the circumvallate papillae [174,175]. *Lgr5/6* cells give rise to all taste bud cell types [175]. The absence of *Neurog2* leads to reduced *Sox2* expression [145,176] and disrupts taste bud formation beyond a limited differentiation of small, single taste buds [163]. Taste bud cells that transduce bitter express *Eya1* suggesting that it may be involved in the differentiation of this cell type [144]. Similarly, the differentiation of type II cells is dependent on the transcription factor, *Pou2f3*, while type III cells depend on *Ascl1* for their differentiation [16,144,177,178,179].

In summary, the development of taste buds is controlled by a series of gene expression events. Once the axons of peripheral neurons reach the taste epithelium, these two cell types become interdependent. Thus, the formation of the peripheral taste system represents the interaction between early and late genes that regulate cell fate and the trophic interactions that occur between taste buds and nerve fibers.

## 7. Development of Neuronal Genes Are Needed for Ears and Epibranchial Placodes

A set of unique genes is characterized so that it requires the vestibular, spiral and epibranchial ganglion neurons [6,16].

**Figure 5:**
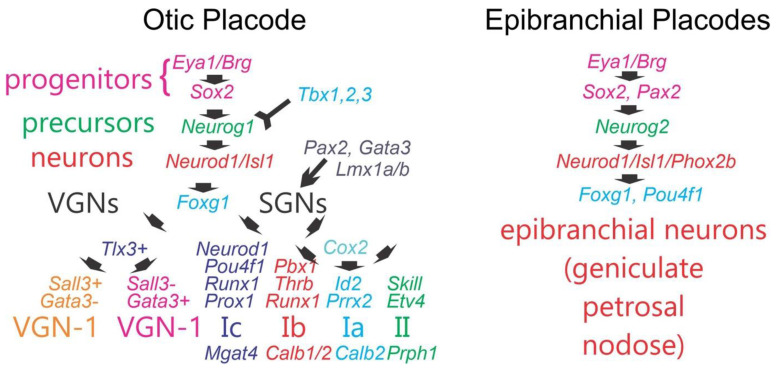
Progenitors of mammals are dependent by initial *Eya1*, *Sox2* and *Neurog1* that are needed for several genes to differentiate neurons of either the otocyst or the epibranchial placodes. In the ear, downstream are a large set of genes, among them are *Neurod1*, *Isl1* and *Pou4f1*. A split is dependent on *Tlx3* that forms VGNs, whereas SGNs are not dependent on *Tlx3*. After that, the upregulation of *Sall3* is needed to make two different kinds of VGNs but has a third population that is unclear as to its origin. SGNs split into four populations: Ia, Ib, Ic and II. A unique set of genes and their expression needs to be verified to develop each type of SGN, notably *Pax2*, *Gata3* and *Lmx1a/b*. For the epibranchial neurons, unique common genes are deviate in the epibranchial neurons by switching the *Neurog1* into *Neurog2* and have early expressions of *Phox2b*, *Isl1*, *Neurod1* and *Foxg1*. Image is compiled with permission from Refs. [4,17,108,116,142,180,186].

Vestibular ganglion neurons (VGNs) and spiral ganglion neurons (SGNs) are independently derived from a common origin, *Eya1*, *Sox2* and *Neurog1* (Figure 5). *Eya1* and *Sox2* define SGN precursor populations, whereas *Neurog1* is needed to initiate the proliferation and differentiation of SGNs [4]. Without *Eya1*, *Sox2* or *Neurog1,* no sensory neurons would develop [4,108]. Downstream there is a segregation of genes that are *Tlx3*-positive for vestibular neurons but are negative for SGN [142]. Furthermore, *Sall3* is in part positive for certain VGNs and negative for other VGNs [180]. 

The diversity of two types of SGNs has been defined: *Neurod1*, *bHLHe22*, *Pou4f1*, *Runx1*, *Prox1* and *Gata3* determines the type Ic (Figure 5). The expressions of *Tshz2*, *Grhl1*, *Rxrg*, *Tie4* and *Id1* are needed for type Ia (*Runx1*), Ib (*Gata3*) and type II (*Etv4*, *Prrx2*) SGNs [116]. There is the upregulation of *Pou4f1*, *Lypd1* and *Mgat4c* (Type 1c), *Lypd1*, *Pou4f1* and *Calb1/2* (Type Ib), *Calb1*, *Pcdg20* and *Prph1* (Type Ia) and *Plk5*, *Prph1* and *Th* [Type II; [116,151]]. In addition, the *Nhlh1/2* and *Isl1* are interacting with *Neurod1* to regulate SGNs [181]. VGN formation [180] requires at least three populations that will develop into the bouton terminal, the calyx terminals, and the mixed terminals (calyx and boutons; [182]).

Several selective genes can selectively induce losses of distinct SGNs. Conditional deletion of *Gata3*, *Lmx1a/b*, *Dicer*, *Shh* or *Pax2* results in the complete loss of SGNs (Figure 5), while many VGNs develop despite the loss of SGNs [52,85,93,183,184,185]. Furthermore, the cochlea is reduced into a sac without forming a cochlear duct in several mutants [52,85,93].

A unique set of *Tbx1*, *2* and *3* deletions interact with *Neurog1* to downturn VGNs and SGNs [142]. *Tbx1* acts as a selector gene which controls neuronal fate in the otocyst. Ablation of *Tbx2* leads to cochlear reduction, but it is unclear how many SGNs form. Likewise, absence of *Tbx3* results in vestibular malformations that require the distribution of VGNs. Combined deletion of both *Tbx2/3DKO* results in incomplete and largely absent cochlear and vestibular structures in newborn mice for which we have no differentiation of VGNs and SGNs.

Downstream are neurotrophins that are needed for sensory neuron development and maturation [187]. The loss of all SGNs is documented for *TrkB* and *TrkC* that signal for *Bdnf* (*TrkB*) and *Ntf3* (*TrkC*). There is an incomplete deletion in the basal cochlear turn that is dependent on *Ntf3* and *TrkC,* while the loss of *Bdnf* and *TrkB* resulted in the reduction and loss of the apex SGNs: *Bdnf* depends on 95% of VGNs while only about 5% are lost in the SGN, mainly in the apex. In contrast, *Ntf3* (*NT3*) is nearly lost of 95% of SGNs, has lost all basal turn in SGNs, but has only an additional loss of about 5% of VGNs [188].

Proliferation starts from E9–14 for VGNs and from E10.5–12.5 for SGNs. The VGNs delaminate and migrate to form a ganglion outside of the ear while the SGNs stay inside the cochlea. Developing neurons reach out first from VGNs at E10; whereas, the first SGNs fibers reach the auditory nuclei at E12.5. Deletion of certain genes [*Neurod1*, *Isl1*; Refs. [112,181]] as well as loss of Schwann cells after *Sox10* or *ErbB2* deletion show a migration of SGNs that mixed with the VGNs [125]. 

In summary, a set of genes is needed for the formation of the SGNs and depends on their development which diversifies into four SGN types. 

Like we detailed for the origin of the otocyst, gustatory ganglion formation depends on the sequential expression of specific genes, resulting in epibranchial placode formation, delamination, migration and cellular differentiation [16]. Initial placode development depends on the expression of the transcription factor *Foxi3*, which is essential for the development of the ear and epibranchial placodes (Figure 5). In the absence of *Foxi3* expression, the ectoderm fails to thicken, and all placodes fail to form [48]. *Six1/2/4* and *Eya1/2* are essential for the specific formation of epibranchial placodes [33,72,126] downstream of *Foxi3.* The *Sox2*, which is ubiquitously expressed in the epibranchial placodes, is required for neuron development [108]. Epibranchial placodes are patterned into rostral and caudal domains by *Notch* signaling [189], which is regulated by *Eya1* [190]. From the *Sox2*+ precursor pool, placode cells divide into a non-neural population and neuroblasts defined by the transcription factor *Neurog2* in mammals [190]. *Neurog2* is critical for neuronal development [163,191], and *Neurog1* plays a similar role in chickens [128,192]. The activation of *Notch* signaling discourages neuronal fate [190] in favor of a non-neuronal cell fate. Downstream of *Neurod1*, *Isl1*, *Pou4f1* and *Phox2b* interact to regulate neuronal migration and differentiation [128,163,193]. Although all neuroblasts express the pro-neural transcription factor *Isl1*, *Phox2b* is required specifically for a visceral sensory neuron (taste) fate [194]. *Foxg1*, *Phox2a* and *Phox2b* are expressed in the placodes during delamination [128], followed by the set of genes *Coe1*, *Drg11* and *Dcx*, which are only activated after the migrating cells have left the placode [128,192]. Once the final position is reached (facial, glossopharyngeal, vagus ganglion), the neurons grow a single process that branches to innervate the targeted taste bud cells and sends the proximal innervation to reach distinct areas of the hindbrain to reach out the solitary tract [16,127,195].

In summary, we provide an overview of otic and epibranchial neuron formation from the earliest expression of precursor cells to the differentiation of vestibular, spiral and epibranchial neurons. 

## 8. Concluding Remarks

A detailed characterization of the human embryonic and fetal inner ear development is important. Specifically, induction and the early morphogenesis of the inner ear require signaling cues deployed in both a spatially and temporally restricted pattern. Mimicking these signals under a chemically defined 3D growth system has been shown in many exciting studies to differentiate stem cells into “inner ear organoids” containing sensory epithelia and neurons. However, it should not be concluded that these neurons that differentiated in inner ear organoids only belong to the otic placode lineage. For instance, many of these neurons might resemble cells in the hindbrain or epibranchial placode lineage. Thus, a careful characterization of stem cell-derived neurons in organoid and 3D cell culture systems, principally regarding their specific lineage identities, is still unsatisfactory. 

The last several years, developments, although promising for the derivation of human inner ear neurosensory cells from stem cells, emphasize the importance to further improve and build on our deep understanding of inner ear induction and early organogenesis. This knowledge can be effectively exploited to faithfully recapitulate in vitro the crucial developmental steps leading to otic progenitors and their neurosensory derivatives, which can be used to develop therapies for the human inner ear.
